# Evidence for Water-Borne Transmission of Highly Pathogenic Avian Influenza H5N1 Viruses

**DOI:** 10.3389/fmicb.2022.896469

**Published:** 2022-05-26

**Authors:** Hongbo Zhang, Yanfeng Yao, Yan Li, Jianjun Chen, Ze Chen

**Affiliations:** ^1^State Key Laboratory of Virology, Wuhan Institute of Virology, Chinese Academy of Sciences, Wuhan, China; ^2^Louisiana State University Health Sciences Center, Shreveport, LA, United States

**Keywords:** Dongting Lake, water-borne, HPAIV H5N1, genotyping, phylogenetic analysis

## Abstract

In this study, we isolated 10 H5N1 strains from water samples in Dongting Lake and 4 H5N1 strains from lakeside backyard poultry. These isolates belonged to three distinct clades (clade 2.3.2, 2.3.4, and 7). Phylogenetic analysis showed a diversified genome constellation. The genetic characteristics of some viruses isolated from water samples were extremely similar to those from lakeside poultry. Pathogenic experiments showed that selected represented isolates in this study were highly pathogenic for SPF chickens but had a diversified virulence in mice. The results of our study suggested the potential transmission of avian influenza (H5N1) between the poultry and wild waterfowls and water body around the habitat may play an important role.

## Introduction

The highly pathogenic H5N1 avian influenza virus (HPAIV) was first identified in China in 1996 [A/goose/Guangdong/1/1996 (Gs/GD)] and infected the human population in Hong Kong in 1997, with 18 recorded cases resulting in six deaths (Claas et al., 1998; Subbarao et al., 1998). Since 2003, Gs/GD lineage H5N1 HPAIV infection in humans has been reported in 16 countries, with 860 laboratory-confirmed cases and 454 deaths as of 2018 (WHO, 2018) and has evolved into numerous phylogenetic lineages (e.g., Clades 2.3.2 and 2.3.4) (Smith et al., 2015). Although human-to-human transmission has not been documented, H5N1 HPAIV has the potential to cause an influenza pandemic.

Importantly, H5N1 HPAIV has reported infected migratory waterfowl and may have, thus, been disseminated to previously unaffected regions. During infection, AIVs preferentially localize in the intestinal tracts of migratory waterfowl and are excreted in high concentrations in their feces (Slemons and Easterday, 1978; Webster et al., 1978). Therefore, transmission between wild waterfowl or between wild waterfowl and domestic poultry is thought to occur *via* the fecal-oral transmission route or by the ingestion of contaminated water (Webster et al., 1992). The Dongting Lake wetland is an important overwintering area situated at the East Asian flyway. Tens of thousands of migratory birds reach here every autumn for wintering. Also, backyard poultry including chicken, duck, and goose are commonly raised in this region. Exposure between wild waterfowl and backyard poultry might result in the events of bidirectional transmission of AIVs occurred. In this study, we provide evidence that the introduction of poultry H5N1 viruses into wild waterfowls occurred and water bodies may play an important role.

## Materials and Methods

### Virus Isolation

The influenza virus in water samples was concentrated and isolated according to the method reported by Webster et al. previously (Ito et al., 1995; Khalenkov et al., 2008). A portion of 200 ml of water sample was added with formaldehyde-fixed erythrocytes to a final concentration of 0.1% and, then, set on ice for 1 h, during which the tube was inverted every 15 min to mix up the content, then, centrifuged at 4°C, 5,000 rpm for 5 min. The supernatant was discarded, while the precipitate was re-suspended with 1 ml phosphate-buffered saline (PBS) (pH7.2) and inoculated into SPF embryonated chicken eggs at a dosage of 0.5 ml per egg and incubated at 37°C.

Viruses were isolated from the swabs of domestic birds by inoculating into SPF embryonated eggs too. And all the isolated viruses were purified by 3 rounds of limiting dilution cloning in SPF embryonated chicken eggs.

### RNA Extraction and Nucleotide Sequencing

The viral genomic RNA was extracted from the allantoic fluids by lysing the viruses with Trizol LS reagent (Life Technologies, Inc.). All segments of the viral genomes were amplified by reverse transcription-PCR (Hoffmann et al., 2001; Li et al., 2007) and then sequenced. The sequence data were edited and aligned by BioEdit version 7.0.5.2.

### Pathogenicity Study

Groups of ten 6-week-old SPF (Specific pathogen Free) white Leghorn chickens were tested according to the recommendation of the Office International des Épizooties (OIE) and the intravenous pathogenicity index (IVPI) of the isolates in chickens was determined.

Groups of eight 6-week-old female BALB/c mice were inoculated intranasally with 10^6^.^5^ EID_50_ of the selected viruses in a volume of 50μl under anesthesia. On day 5 after inoculation, three inoculated mice were sacrificed, and organs were collected for virus titration. The remaining mice were monitored daily for weight loss and mortality. The mouse 50% minimal lethal doses (MLD_50_) of the viruses were determined by inoculating 6 groups of mice (*n* = 5 mice each) with 10-fold serial dilutions of the virus. The MLD_50_ was calculated by the method of Reed and Muench.

The virus isolation and the experiments of pathogenicity study and other experiments related to living viruses were all conducted in Biosafety Level 3 facilities.

## Phylogenetic Analysis

HA gene segments of 14 isolates in Dongting Lake and H5 Clade reference sequences were used as datasets to classify HA clade and corresponding NA sequences of these strains were chosen as datasets for NA phylogenetic analysis. All influenza A virus sequences available in National Center for Biotechnology Information (NCBI) and the Global Initiative on Sharing All Influenza Data (GISAID) were downloaded and integrated into one database. For each internal gene, 14 sequences of viruses in Dongting Lake were queried against the integrated database by running the BLAST program locally with default parameters and the top 100 hits were collected. Sequences with the same accession number were removed and the rest sequences were as internal gene datasets.

The alignment of each dataset was generated with Clustal Omega version 1.2.1 with default parameters. Maximum likelihood phylogenetic trees were calculated with software RAxML version 8.2.10^1^ under the GTRGAMMA model with 1,000 bootstraps.

## Results and Discussion

As part of routine surveillance in Dongting Lake, 963 water samples were collected from areas where large number of wild waterfowls aggregated, between March 2007 and December 2008. Meanwhile, 806 cloacal swab samples were also collected from lakeside backyard poultry in this region. For water samples, 40 mL of surface water was collected in a sterilized 50-mL screw-cap plastic vial. Both water samples and swab samples were stored in the portable refrigerator and sent to a laboratory, then stored at −80°C until assayed. The water samples were treated according to the method reported previously (Ito et al., 1995; Khalenkov et al., 2008). Then, the treated water samples and swabs were inoculated into SPF embryonated eggs. A total of 10 H5N1 viruses were successfully recovered from the water samples. Meanwhile, 4 viruses were isolated from backyard poultry (Supplementary Table 1). Either in 2007 or 2008, H5N1 viruses were isolated from both water and swab samples. We sequenced full-length genomes and found the polybasic amino acid sequence, RRRKR*GL in the hemagglutinin (HA) cleavage site, confirming the virus can be classified as highly pathogenic. Sequences of those 14 H5N1 influenza isolates were deposited into the Genbank database under accession nos. GU182139-GU182250.

**FIGURE 1:**
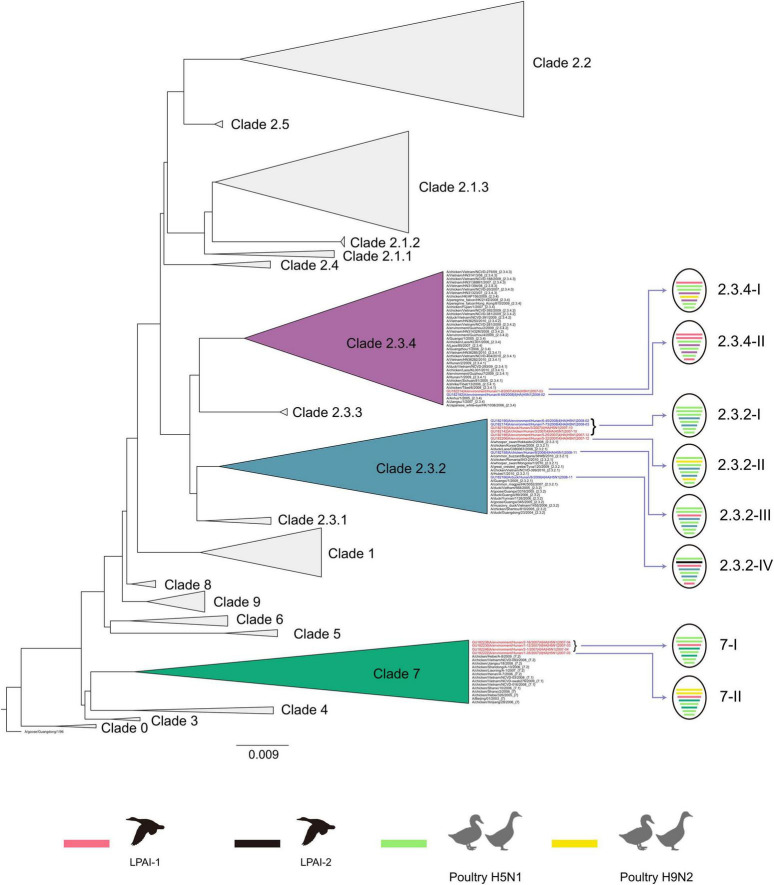
HA Clade and Genotype of isolates in Dongting Lake. Viruses isolated from Dongting Lake in 2007 and 2008 are colored in red and blue, respectively. Genotype of virus are shown in the format of colored ovals with lines that represents the eight genes (from top to bottom: PB2, PB1, PA, HA, NP, NA, MP, and NS). Internal gene segments are colored based on their origin, while HA and NA gene segments are colored according to the HA Clade. Details of phylogenetic trees are available in Supplementary Figure 1.

To understand the genetic relationship of H5N1 viruses with other viruses, we carried out a phylogenetic analysis of H5N1 relevant sequences from public databases. In the HA phylogenetic tree, the 14 Dongting Lake H5N1 viruses fall into 3 different sublineages, and they were clade 2.3.2 (*n* = 8), clade 2.3.4 (*n* = 2), and clade 7 (*n* = 4), respectively (Figure 1 and Supplementary Figure 1A). Analysis of the NA gene of the isolates showed a similar phylogenetic pattern to the HA gene (Supplementary Figure 1B). The result also showed that clade 2.3.2 and 2.3.4 strains were isolated both in 2007 and 2008, and clade 7 strains were only recovered in 2007. As for 10 H5N1 strains recovered from water in 2007 and 2008, they covered all those three clades.

Phylogenetic analyses of the internal genes indicate the diversity of H5N1 viruses in Dongting Lake (Figure 1 and Supplementary Figures 1C–H). According to the sublineages of internal genes, both clade 7 and clade 2.3.4 have 2 distinct genome constellations and they were genotype 7-I (*n* = 3) and 7-II (*n* = 1), and genotype 2.3.4-I (*n* = 1) and 2.3.4-II (*n* = 1), respectively. The genotype 7-II differed from 7-I in the origin of PB2 and PB1 genes, with both genes of 7-II originating from poultry H9N2 viruses. The genotypes 2.3.4-I and 2.3.4-II have a different origin in the PB1, NP, and NS genes, the PB1 and NS genes of genotype 2.3.4-II were acquired from wild aquatic bird’s gene pool. As for clade 2.3.2, eight strains diversified into 4 genotypes and they were 2.3.2-I (*n* = 5), 2.3.2-II (*n* = 1), 2.3.2- III (*n* = 1) and 2.3.2-IV (*n* = 1). Gene segments from poultry H9N2 viruses and wild aquatic bird gene pools were also observed to contribute to the generation of various genotypes of clade 2.3.2. Phylogenetic analysis suggested various clades and distinct genotypes of H5N1 viruses were introduced into Dongting Lake, from 2007 to 2008.

A potential transmission event, from lakeside poultry to Dongting Lake, may occur in 2007. In October 2007, two genotype 2.3.2-I strains were isolated from chicken and duck at a lakeside backyard [A/Chicken/Hunan/3/2007(H5N1), and A/Duck/Hunan/3/2007(H5N1)]. Two months later, a highly similar strain with the same genotype was recovered from a water sample in a lake [A/Environment/Hunan/5-25/2007(H5N1)]. In the following 2008 Spring, this genotype virus was again recovered from water samples in the lake [A/Environment/Hunan/6-45/2008(H5N1), A/Environment/Hunan/7-73/2008(H5N1)]. Since the AIVs can maintain an infectious state for less than one month and the genotype 2.3.2-I was recovered from water for more than 2 months, this result highly suggested that this virus may circulate in wild aquatic birds during their wintering time.

Molecular characterization showed the receptor-binding pocket of the HA gene in the isolates in this study all contained QSG amino acid residues (Supplementary Table 2), indicating that the virus preferred binding to the avian-like receptor (Ha et al., 2001). At the stalk of the NA gene, all the isolates had 49-68 amino acid deletion, which might be necessary for the adaptation of the virus from wild birds to domestic fowls (chicken) (Matrosovich et al., 1999). No amino acid substitution, such as E627K or D701N in PB2, which was considered might enhance the virulence of the virus in mammals (Hatta et al., 2001; Li et al., 2005), was observed in any isolates in this study. The amino acid substitution from S to N at site 31 of the M2 gene was observed in six viruses isolated in this study, which might result in the resistance of the virus to amantadine and rimantadine (Suzuki et al., 2003). The deletions of amino acids at sites 80-84 of the NS1 gene were observed in all the isolates except A/Environment/Hunan/6-69/2008(H5N1), which was considered might enhance the virulence of the virus (Long et al., 2008).

We further determined the pathogenicity of selected stains in SPF chickens and mice (Supplementary Table 3). All the selected viruses are high-pathogenic in chickens and had an IVPI of 3, while the pathogenicity of these viruses in mice was highly diverse. Four strains, including genotype 2.3.4-I, 2.3.2-I, and 2.3.2-II showed high-pathogenic and caused systemic infection in mice, with viruses detected in multiple organs including brain tissue. While three strains, including genotype 7-I, 7-II, and 2.3.4-II only replicated in the lung or spleen and showed low-pathogenic in mice.

In summary, we isolated 10 H5N1 strains from water samples in Dongting lake and 4 H5N1 strains from lakeside backyard poultry. These isolates belonged to 3 distinct clades (clade 2.3.2, 2.3.4, and 7). Phylogenetic analysis showed a diversified genome constellation. The genetic characteristics of some viruses isolated from water samples were extremely similar to those from lakeside poultry. To confirm that the water isolates have the same origin as the domestic isolates, we selected the chicken and duck viruses isolated in October 2007 and the two viruses isolated in December 2007 for the homology analysis. The nucleotide sequences for each gene segment of the isolated viruses were aligned with ClustalW. The results showed that all the 8 gene segments of the H5N1 isolated from chicken were highly homologous to those of the virus isolated from duck in the present study. Among them, the homology of HA, PA, NP, and NS is as high as 99% or more. In addition, the NA, PB2, PB1, and M genes also have a very high homology range from 96.3 to 97.5%. The high homology of the relevant genes between the chicken isolate and duck isolate suggested that the chicken and duck were infected with the same H5N1 virus. On the other hand, all the 8 gene segments from the domestic poultry isolates have a great similarity to relevant segments of the water isolates. In detail, the NA, PB2, PB1, and NS gene of A/Chicken/Hunan/3/2007(H5N1) have the 99% homology to the A/Environment/Hunan/5-25/2007(H5N1) or A/Environment/Hunan/5-32/2007(H5N1). The HA, PA, NP, and M gene also have 95.3-97.4% homology, as shown in Supplementary Table 4. Similarly, the relevant eight gene segments of the duck isolate and water isolates have 95.4 to 99.9% homology (Supplementary Table 4). These results suggested that the water strains had great genetic similarity with the strains isolated from backyard poultry and the water isolates and domestic poultry isolates probably originated from the same virus. Pathogenic experiments showed that selected represented isolates in this study were highly pathogenic for SPF chickens but had a diversified virulence in mice.

In Dongting Lake wetland, the mixed breeding of chicken, duck, and goose is very popular on the lakeside, so, that the domestic ducks and the wild aquatic birds can share the same water body. Since several water strains had great genetic similarity with backyard poultry strains that were isolated several months before, this provided evidence that dissemination of the AIVs between the backyard poultry and the wild aquatic birds in this region occurred. This result highly suggested that the water body might play an important vector in circulation and spread of AIVs. However, the results of this study are not fully direct evidence of the introduction of poultry H5N1 viruses into wild waterfowls. Because the possibility that waterfowl carrying an HPAIV of a specific genotype disseminated the virus both to poultry birds and to water habitat was not ruled out. Dongting Lake is an important habitat and overwintering area for migratory birds, surveillance here will likely yield the first evidence of the virus introduction and is helpful in taking more effective measures to prevent and control AIVs.

## Data Availability Statement

The datasets presented in this study can be found in online repositories. The names of the repository/repositories and accession number(s) can be found in the article/Supplementary Material.

## Ethics Statement

The animal study was reviewed and approved by Laboratory Animal welfare committee, Wuhan institute of virology, CAS.

## Author Contributions

HZ collected the samples, isolated the viruses, sequenced all of the isolated viruses, and did all of the animal experiments. YY and YL collected the samples and isolated the viruses. JC and ZC edited the manuscript. All authors contributed to the article and approved the submitted version.

## Conflict of Interest

The authors declare that the research was conducted in the absence of any commercial or financial relationships that could be construed as a potential conflict of interest.

## Publisher’s Note

All claims expressed in this article are solely those of the authors and do not necessarily represent those of their affiliated organizations, or those of the publisher, the editors and the reviewers. Any product that may be evaluated in this article, or claim that may be made by its manufacturer, is not guaranteed or endorsed by the publisher.

## Footnotes

1
https://www.phylo.org/
