# Supplementary material for: Evidence for Water-Borne Transmission of Highly Pathogenic Avian Influenza H5N1 Viruses
Source: Front Microbiol. 2022 May 26;13:896469. doi: 10.3389/fmicb.2022.896469 (PMC9183062; doi:10.3389/fmicb.2022.896469)
Supplement: Supplementary file 2 [file Table_2.docx]

Supplementary Table S2. Molecular characterization of HA, NA, M2, PB2, and NS1 at representative sites

| Stain name | Genotype | HA | | NA* | | M2 | | PB2 | | NS1* | | | |
| --- | --- | --- | --- | --- | --- | --- | --- | --- | --- | --- | --- | --- | --- |
|  |  | Cleavage site | RBS | 49-68 | 274 | 26 | 31 | 627 | 701 | 80-84 | 42 | 92 | 149 |
| A/Environment/Hunan/1-8/2007(H5N1) | 2.3.4-I | E-RRRKR/G | QSG | del | H | L | S | E | D | del | S | D | A |
| A/Environment/Hunan/1-12/2007(H5N1) | 7-I | EGRRRKR/G | QSG | del | H | L | N | E | D | del | S | D | A |
| A/Environment/Hunan/1-35/2007(H5N1) | 7-II | EGRRRKR/G | QSG | del | H | L | N | E | D | del | S | D | A |
| A/Environment/Hunan/2-1/2007(H5N1) | 7-I | EGRRRKR/G | QSG | del | H | L | N | E | D | del | S | D | A |
| A/Environment/Hunan/2-16/2007(H5N1) | 7-I | EGRRKKR/G | QSG | del | H | L | N | E | D | del | S | D | A |
| A/Chicken/Hunan/3/2007(H5N1) | 2.3.2-I | E-RRRKR/G | QSG | del | H | L | S | E | D | del | S | D | A |
| A/Duck/Hunan/3/2007(H5N1) | 2.3.2-I | E-RRRKR/G | QSG | del | H | L | S | E | D | del | S | D | A |
| A/Environment/Hunan/5-25/2007(H5N1) | 2.3.2-I | E-RRRKR/G | QSG | del | H | L | S | E | D | del | S | D | A |
| A/Environment/Hunan/5-32/2007(H5N1) | 2.3.2-II | E-RRRKR/G | QSG | del | H | L | S | E | D | del | S | D | A |
| A/Environment/Hunan/6-45/2008(H5N1) | 2.3.2-I | E-RRRKR/G | QSG | del | H | L | N | E | D | del | S | D | A |
| A/Environment/Hunan/6-69/2008(H5N1) | 2.3.4-II | E-RRRKR/G | QSG | del | H | L | S | E | D | No | S | D | A |
| A/Environment/Hunan/7-73/2008(H5N1) | 2.3.2-I | E-RRRKR/G | QSG | del | H | L | N | E | D | del | S | D | A |
| A/Chicken/Hunan/8/2008(H5N1) | 2.3.2-III | E-RRRKR/G | QSG | del | H | L | S | E | D | del | S | D | A |
| A/Duck/Hunan/8/2008(H5N1) | 2.3.2-IV | E-RRRKR/G | QSG | del | H | L | S | E | D | del | S | D | A |

* del: deletion; No: No deletion.
