# Supplementary material for: Evidence for Water-Borne Transmission of Highly Pathogenic Avian Influenza H5N1 Viruses
Source: Front Microbiol. 2022 May 26;13:896469. doi: 10.3389/fmicb.2022.896469 (PMC9183062; doi:10.3389/fmicb.2022.896469)
Supplement: Supplementary file 3 [file Table_3.DOCX]

| Selected strains | Genotype | Pathogenicity in Chicken | | Virus titers in organs of mice  (log_10_ EID_50_/ml ±SD)† | | | | |
| --- | --- | --- | --- | --- | --- | --- | --- | --- |
|  |  | Inoculation dose  (log_10_EID_50_) | IVPI * | Lung | Spleen | Brain | Kidney | MLD_50_(logEID_50_)‡ |
| A/Environment/Hunan/1-8/2007(H5N1) | 2.3.4-I | 7.0 | 3.0 | 7.0±0.3 | 4.5±0.9 | 4.6±1.0 | 3.7±0.3 | 1.2 |
| A/Duck/Hunan/3/2007(H5N1) | 2.3.2-I | 6.6 | 3.0 | 6.7±0.2 | 3.0±0.3 | 2.9±1.0 | 2.2±0.8 | 2.5 |
| A/Environment/Hunan/5-32/2007(H5N1) | 2.3.2-II | 7.3 | 3.0 | 7.0±0.3 | 4.7±1.6 | 4.4±0.4 | 4.1±0.4 | 2.2 |
| A/Environment/Hunan/7-73/2008(H5N1) | 2.3.2-I | 7.5 | 3.0 | 7.7±0.3 | 4.4±0.5 | 7.1±0.3 | 5.6±0.4 | 1.5 |
| A/Environment/Hunan/6-69/2008(H5N1) | 2.3.4-II | 6.3 | 3.0 | 4.2±1.0 | － | － | － | >6.5 |
| A/Environment/Hunan/1-12/2007(H5N1) | 7-I | 7.6 | 3.0 | 5.2±0.8 | ＋ | － | － | >6.5 |
| A/Environment/Hunan/1-35/2007(H5N1) | 7-II | 7.3 | 3.0 | 3.6±0.4 | － | － | － | >6.5 |

Supplementary Table S3. Pathogenicity of selected Dongting Lake H5N1 isolates in chickens and mice

*The IVPI was determined according to the recommendations of the Office International des Epizooties.

†Six-week-old BALB/c female mice were infected intranasally with 10^6.5^ EID_50_ of virus in a 50ul volume. Three mice from each group were euthanized on day 5 post-inoculation, and virus in the organs was titrated in eggs. SD, standard deviation; “+” virus was detected only in undiluted samples; “–” virus was not detected.

‡The MLD_50_ dose was determined by inoculating groups of 6-week-old female mice intranasally with 10-fold serial dilutions of each virus according to the Reed and Muench method.
