# Supplementary material for: Evidence for Water-Borne Transmission of Highly Pathogenic Avian Influenza H5N1 Viruses
Source: Front Microbiol. 2022 May 26;13:896469. doi: 10.3389/fmicb.2022.896469 (PMC9183062; doi:10.3389/fmicb.2022.896469)
Supplement: Supplementary file 4 [file Table_4.DOCX]

| GENE | Virus Strain | Ck/HN/3/07 | Dk/HN/3/07 | En/HN/5-25/07 | En/HN/5-32/07 |
| --- | --- | --- | --- | --- | --- |
| HA | Ck/HN/3/07 | 100 | 99.3 | 95.3 | 95.1 |
|  | Dk/HN/3/07 | 99.3 | 100 | 95.9 | 95.7 |
|  | En/HN/5-25/07 | 95.3 | 95.9 | 100 | 99.8 |
|  | En/HN/5-32/07 | 95.1 | 95.7 | 99.8 | 100 |
| NA | Ck/HN/3/07 | 100 | 96.5 | 96.3 | 99.9 |
|  | Dk/HN/3/07 | 96.5 | 100 | 99.6 | 96.5 |
|  | En/HN/5-25/07 | 96.3 | 99.6 | 100 | 96.5 |
|  | En/HN/5-32/07 | 99.9 | 96.5 | 96.5 | 100 |
| PB2 | Ck/HN/3/07 | 100 | 97.1 | 99.2 | 96.7 |
|  | Dk/HN/3/07 | 97.1 | 100 | 96.9 | 99.5 |
|  | En/HN/5-25/07 | 99.2 | 96.9 | 100 | 97 |
|  | En/HN/5-32/07 | 96.7 | 99.5 | 97 | 100 |
| PB1 | Ck/HN/3/07 | 100 | 96.6 | 99.3 | 88.5 |
|  | Dk/HN/3/07 | 96.6 | 100 | 96.7 | 91 |
|  | En/HN/5-25/07 | 99.3 | 96.7 | 100 | 88.3 |
|  | En/HN/5-32/07 | 88.5 | 91 | 88.3 | 100 |
| PA | Ck/HN/3/07 | 100 | 99.7 | 96.5 | 96.8 |
|  | Dk/HN/3/07 | 99.7 | 100 | 96.5 | 96.7 |
|  | En/HN/5-25/07 | 96.5 | 96.5 | 100 | 99.4 |
|  | En/HN/5-32/07 | 96.8 | 96.7 | 99.4 | 100 |
| NP | Ck/HN/3/07 | 100 | 99.9 | 95.3 | 95.4 |
|  | Dk/HN/3/07 | 99.9 | 100 | 95.4 | 95.5 |
|  | En/HN/5-25/07 | 95.3 | 95.4 | 100 | 99.9 |
|  | En/HN/5-32/07 | 95.4 | 95.5 | 99.9 | 100 |
| M | Ck/HN/3/07 | 100 | 97.5 | 97.4 | 87.6 |
|  | Dk/HN/3/07 | 97.5 | 100 | 99.9 | 89.9 |
|  | En/HN/5-25/07 | 97.4 | 99.9 | 100 | 89.8 |
|  | En/HN/5-32/07 | 87.6 | 89.9 | 89.8 | 100 |
| NS | Ck/HN/3/07 | 100 | 99.9 | 99.9 | 99.9 |
|  | Dk/HN/3/07 | 99.9 | 100 | 99.8 | 99.8 |
|  | En/HN/5-25/07 | 99.9 | 99.8 | 100 | 100 |
|  | En/HN/5-32/07 | 99.9 | 99.8 | 100 | 100 |

Supplementary Table S4. Homology (%) of nucleotide sequences of eight genes of virus strains isolated in domestic poultry to themselves and the viruses isolated in the water in the subsequent two months*.

*Ck/HN/3/07: A/Chicken/Hunan/3/2007(H5N1), Dk/HN/3/07: A/Duck/Hunan/3/2007(H5N1), En/HN/5-25/07: A/Environment/Hunan/5-25/2007(H5N1), En/HN/5-32/07: A/Environment/Hunan/5-32/2007(H5N1).
