# Supplementary figures and images for: Evidence for Water-Borne Transmission of Highly Pathogenic Avian Influenza H5N1 Viruses
Source: Front Microbiol. 2022 May 26;13:896469. doi: 10.3389/fmicb.2022.896469 (PMC9183062; doi:10.3389/fmicb.2022.896469)

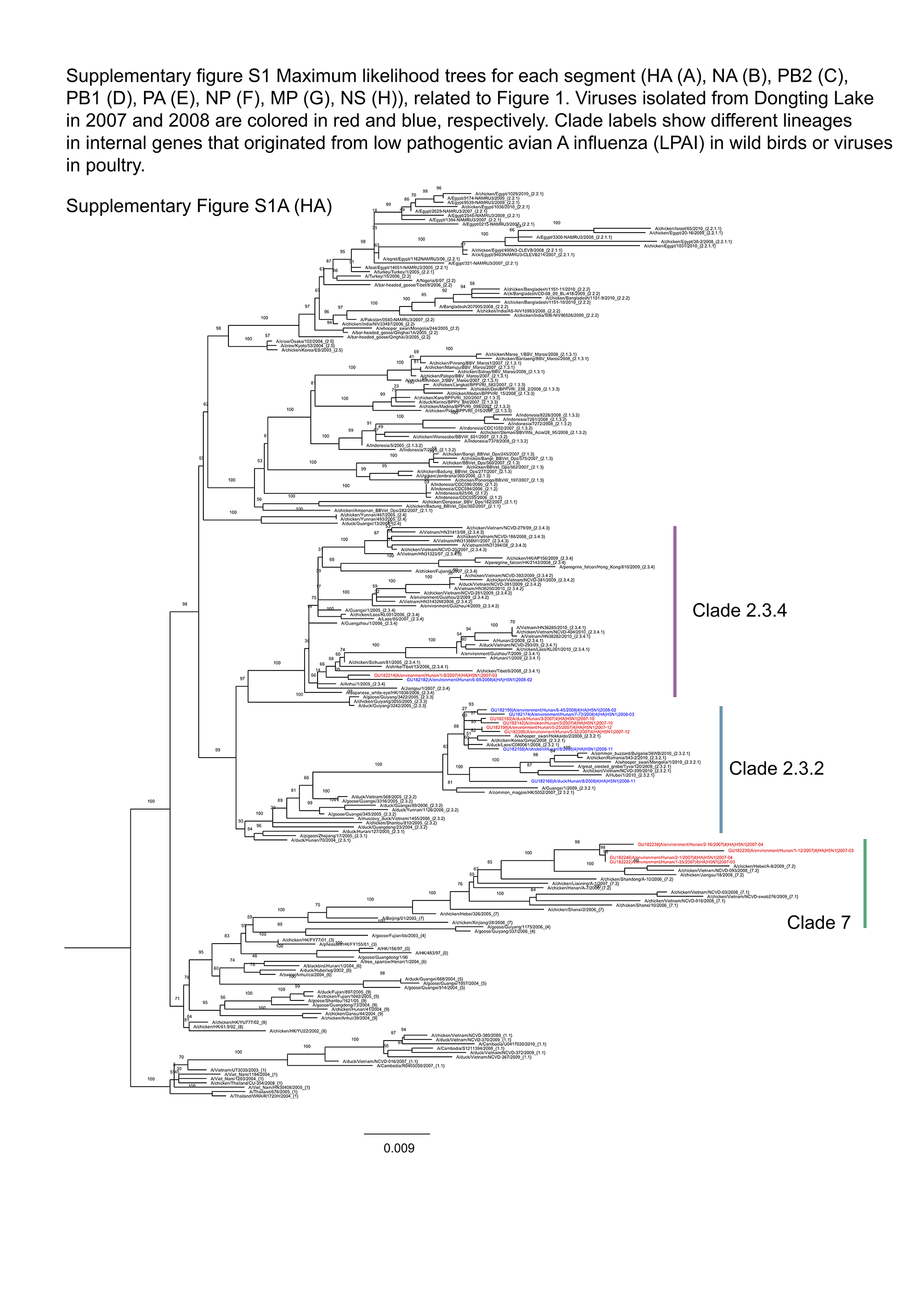

Supplement: Supplementary file 5 [file Image_1.TIF]

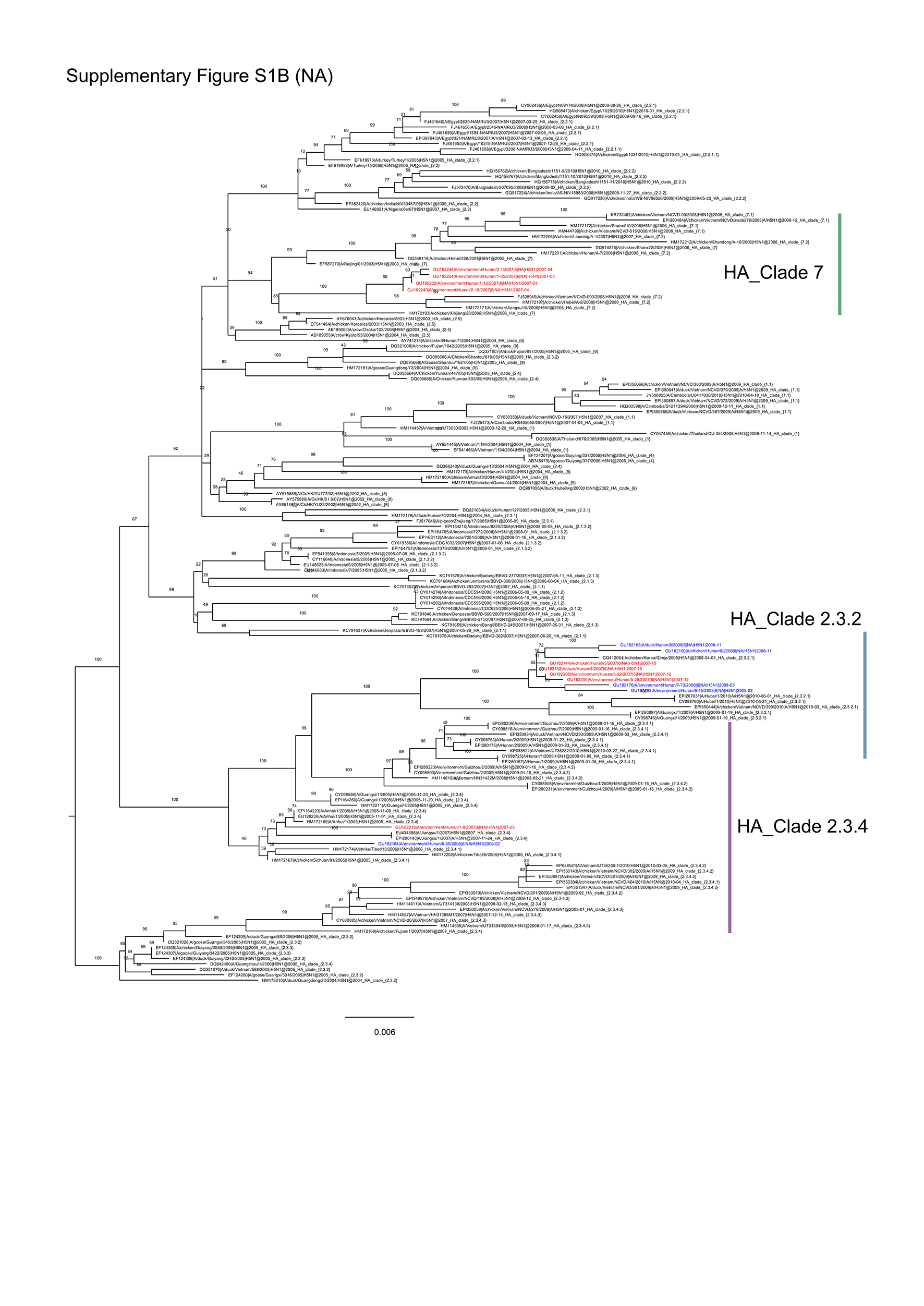

Supplement: Supplementary file 6 [file Image_2.TIF]

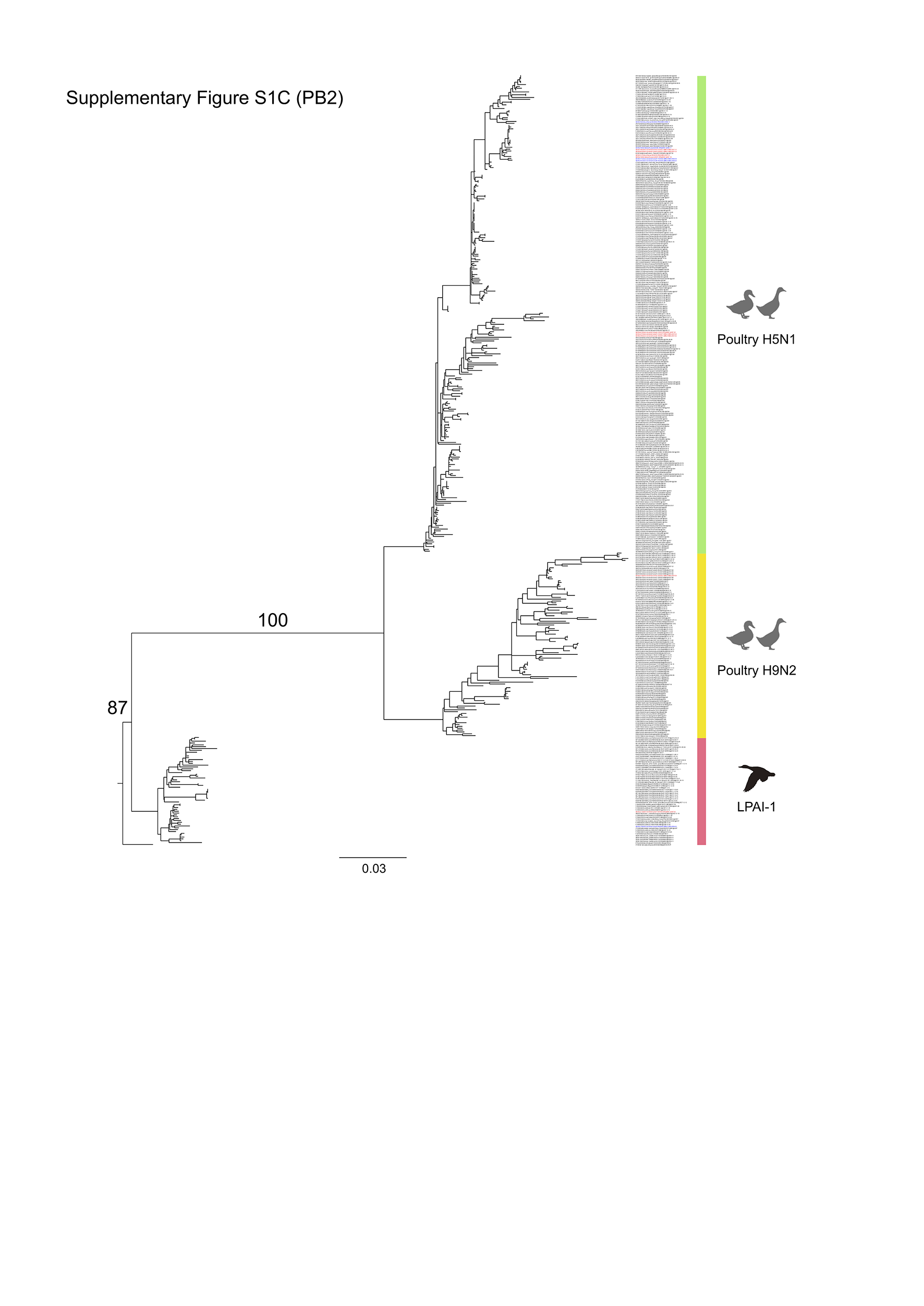

Supplement: Supplementary file 7 [file Image_3.TIF]

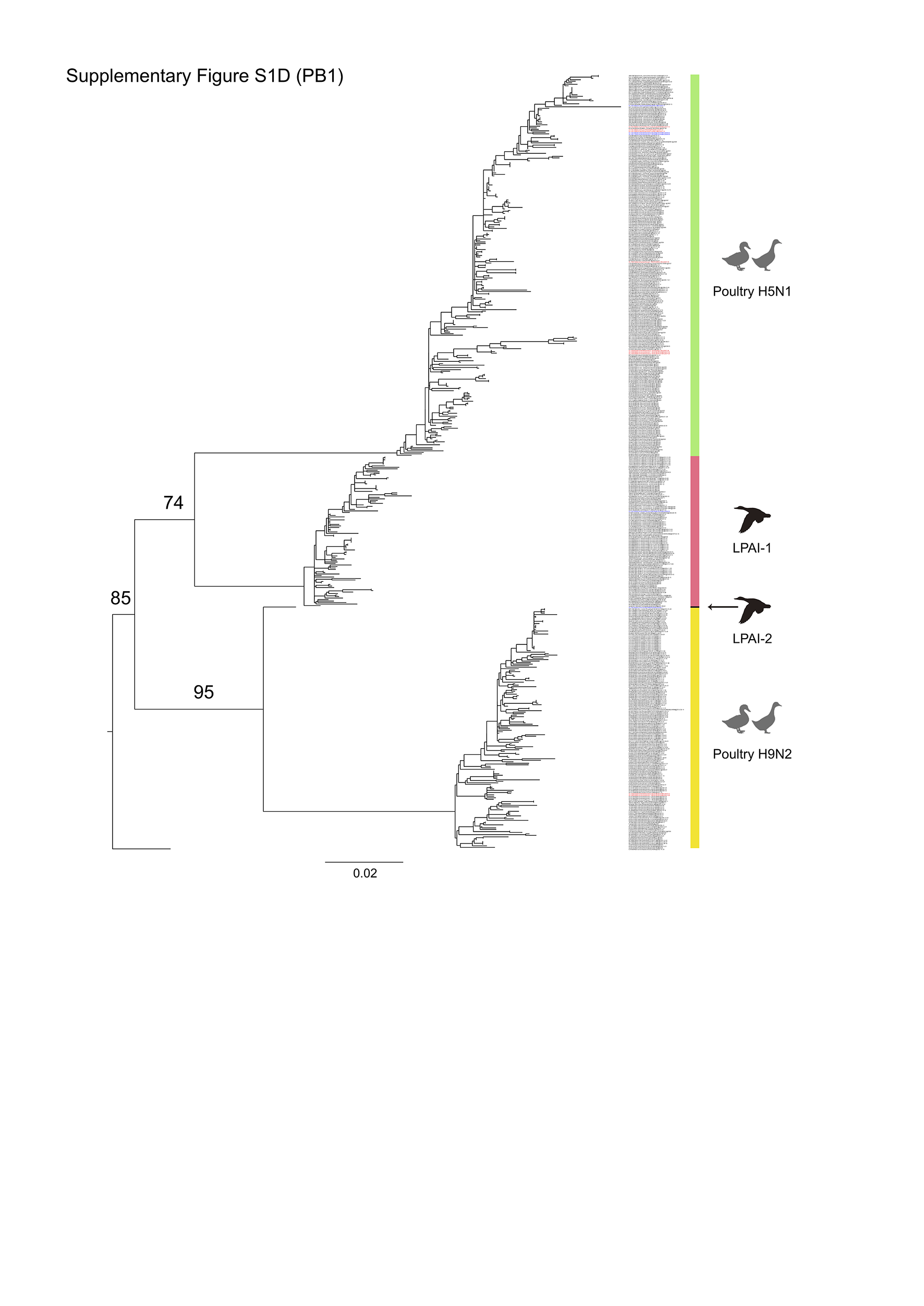

Supplement: Supplementary file 8 [file Image_4.TIF]

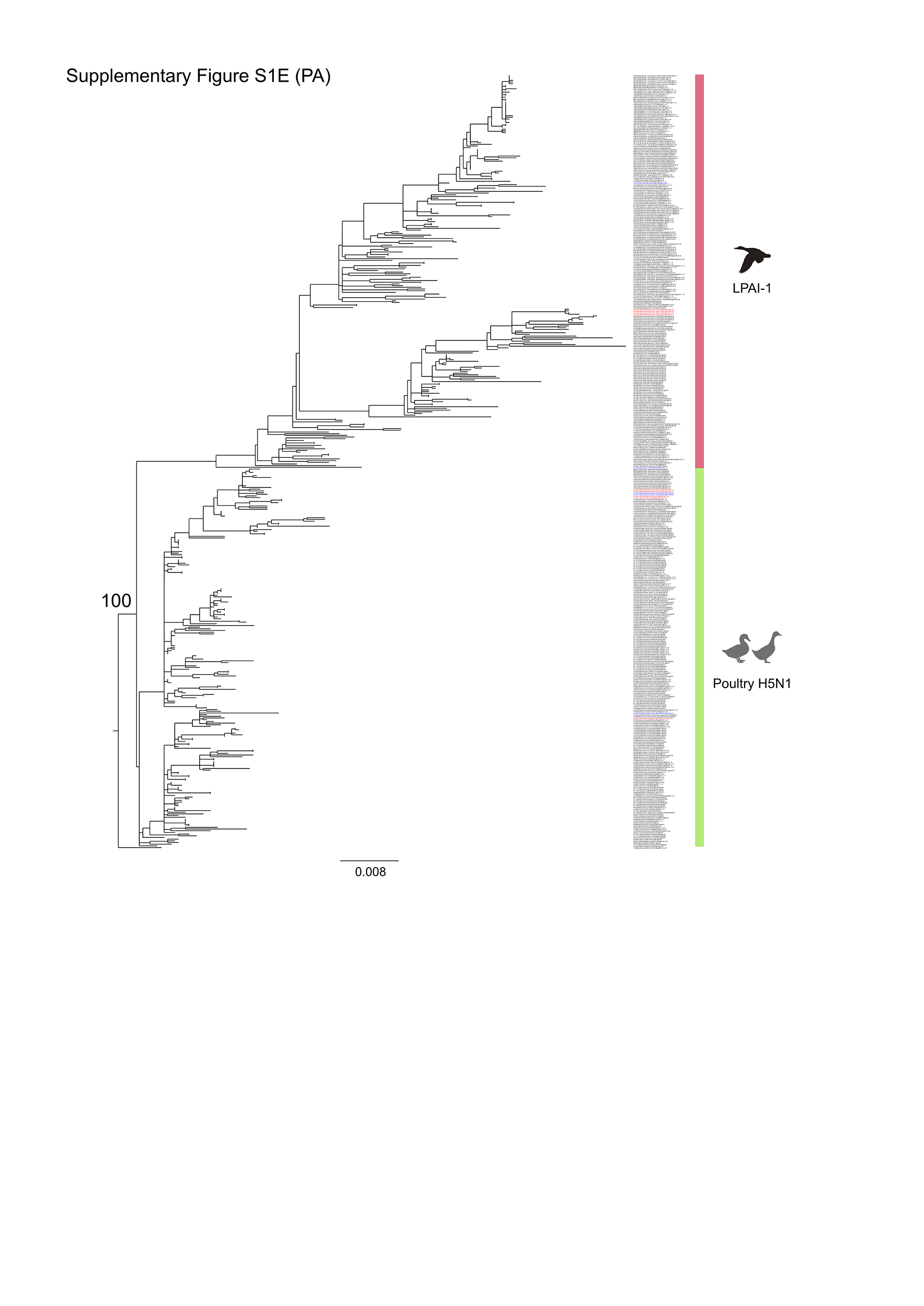

Supplement: Supplementary file 9 [file Image_5.TIF]

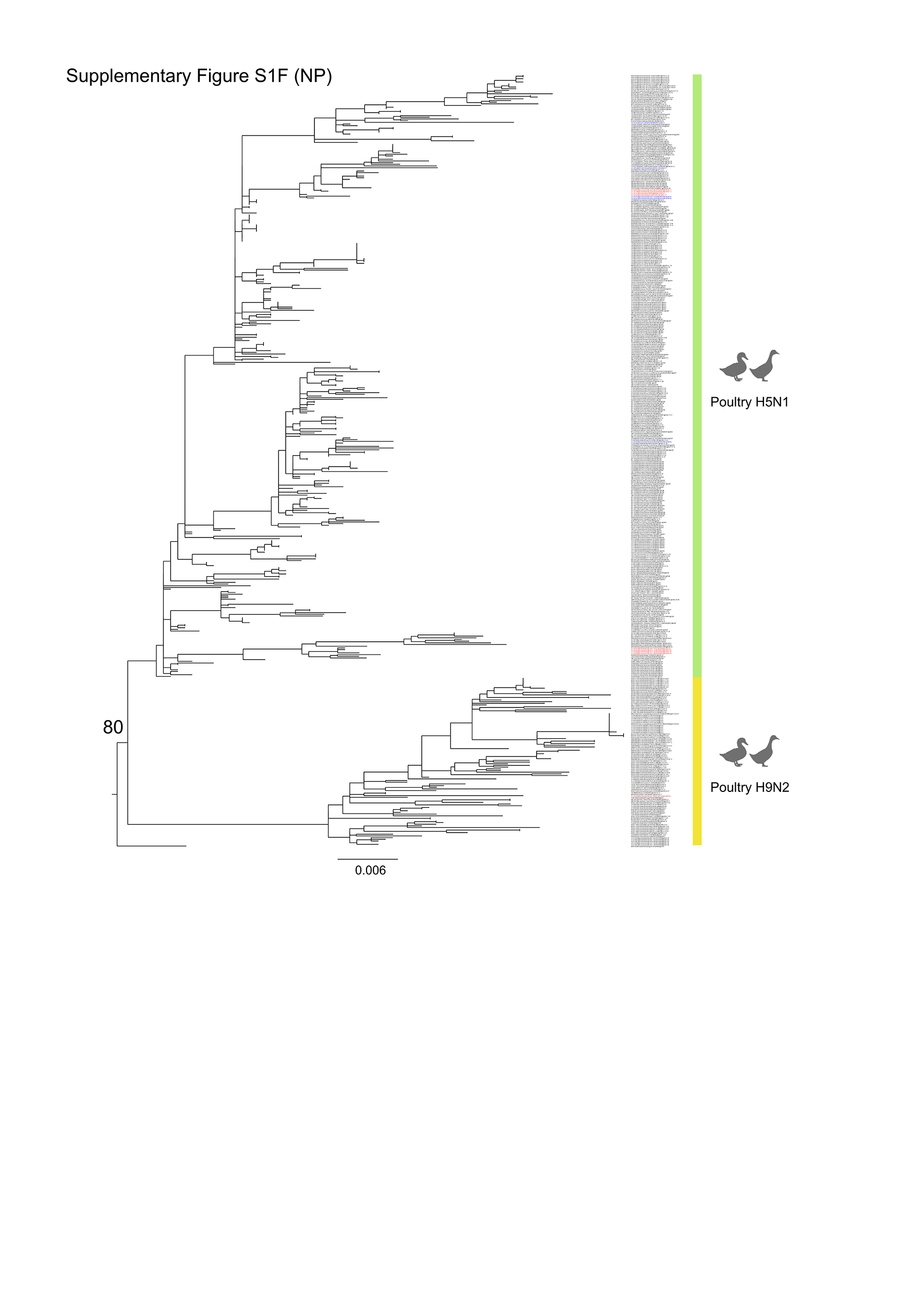

Supplement: Supplementary file 10 [file Image_6.TIF]

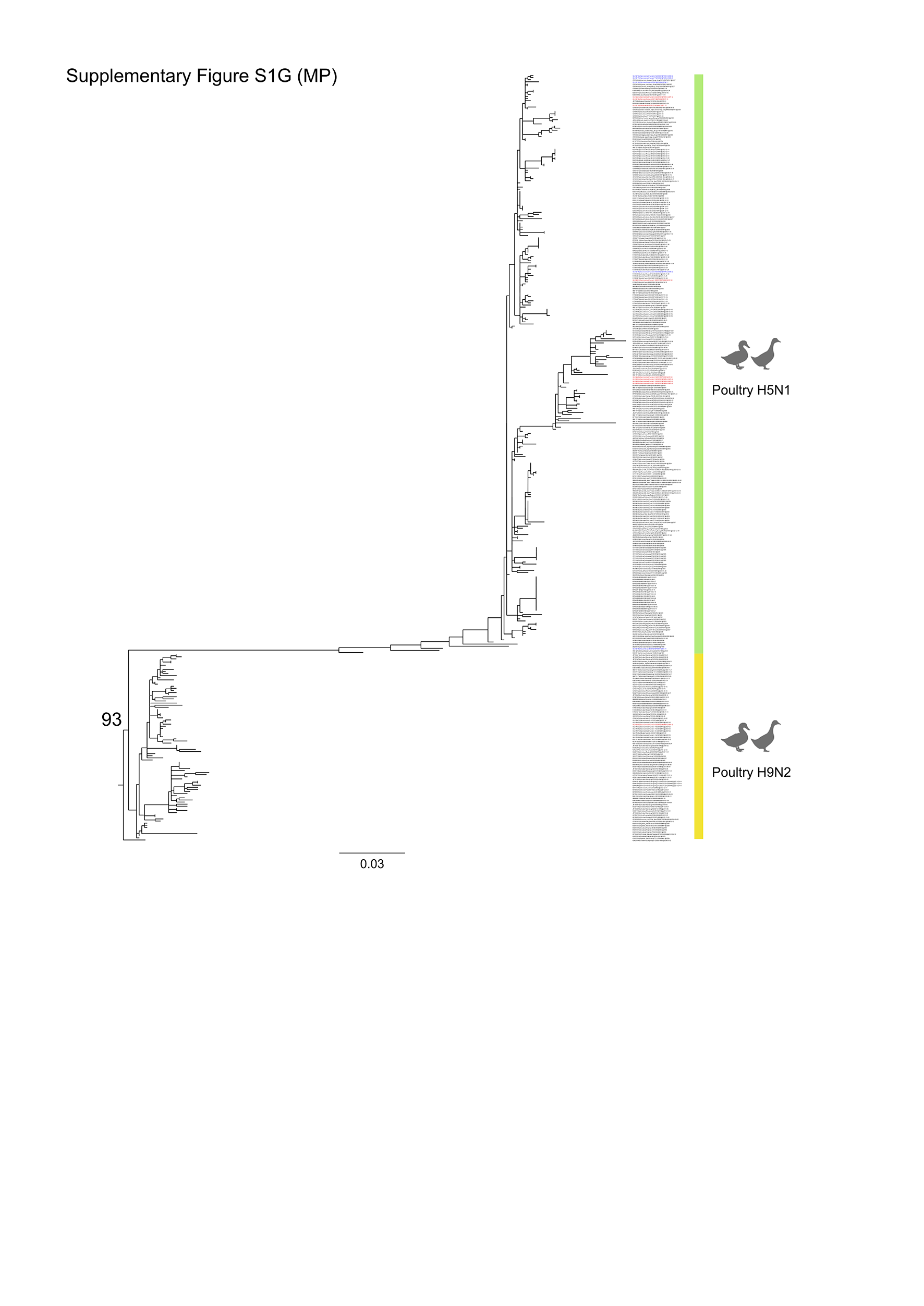

Supplement: Supplementary file 11 [file Image_7.TIF]

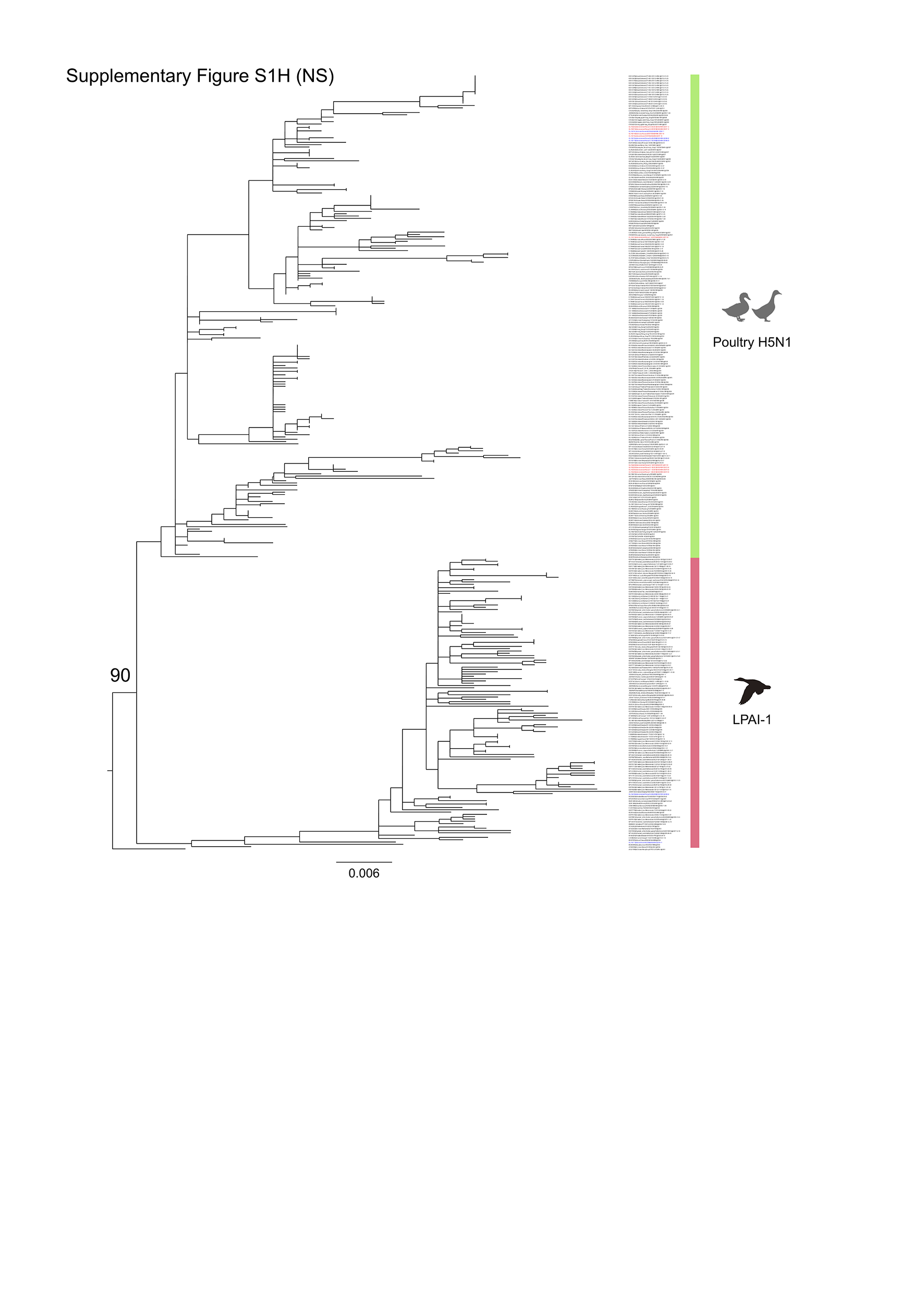

Supplement: Supplementary file 12 [file Image_8.TIF]
